# Ceramides in Autoimmune Rheumatic Diseases: Existing Evidence and Therapeutic Considerations for Diet as an Anticeramide Treatment

**DOI:** 10.3390/nu15010229

**Published:** 2023-01-02

**Authors:** Ioanna Alexandropoulou, Maria G. Grammatikopoulou, Kalliopi K. Gkouskou, Agathi A. Pritsa, Tonia Vassilakou, Eirini Rigopoulou, Helen M. Lindqvist, Dimitrios P. Bogdanos

**Affiliations:** 1 Department of Nutritional Sciences & Dietetics, Faculty of Health Sciences, International Hellenic University, Alexander Campus, GR-57400 Thessaloniki, Greece; 2 Department of Rheumatology and Clinical Immunology, University General Hospital of Larissa, Faculty of Medicine, School of Health Sciences, University of Thessaly, Biopolis, GR-41110 Larissa, Greece; 3 Laboratory of Biology, School of Medicine, National and Kapodistrian University of Athens, GR-11527 Athens, Greece; 4 Department of Public Health Policy, School of Public Health, University of West Attica, GR-11521 Athens, Greece; 5 Department of Medicine and Research Laboratory of Internal Medicine, University Hospital of Larissa, Biopolis, GR-41222 Larissa, Greece; 6 Department of Internal Medicine and Clinical Nutrition, Institute of Medicine, Sahlgrenska Academy, University of Gothenburg, P.O. Box 115, 40530 Gothenburg, Sweden

**Keywords:** sphingosine, sphingolipid, metabolomics, lipidomics, sphingolipidomics, anti-ceramide, metabolomics, rheumatology, immunonutrition

## Abstract

Autoimmune rheumatic diseases (AIRDs) constitute a set of connective tissue disorders and dysfunctions with akin clinical manifestations and autoantibody responses. AIRD treatment is based on a comprehensive approach, with the primary aim being achieving and attaining disease remission, through the control of inflammation. AIRD therapies have a low target specificity, and this usually propels metabolic disturbances, dyslipidemias and increased cardiovascular risk. Ceramides are implicated in inflammation through several different pathways, many of which sometimes intersect. They serve as signaling molecules for apoptosis, altering immune response and driving endothelial dysfunction and as regulators in the production of other molecules, including sphingosine 1-phosphate (S1P) and ceramide 1-phosphate (C1P). With lipid metabolism being severely altered in AIRD pathology, several studies show that the concentration and variety of ceramides in human tissues is altered in patients with rheumatic diseases compared to controls. As a result, many in vitro and some in vivo (animal) studies research the potential use of ceramides as therapeutic targets in rheumatoid arthritis (RA), ankylosing spondylitis, systemic lupus erythematosus, fibromyalgia syndrome, primary Sjögren’s syndrome, systemic sclerosis, myositis, systemic vasculitis and psoriatic arthritis. Furthermore, the majority of ceramide synthesis is diet-centric and, as a result, dietary interventions may alter ceramide concentrations in the blood and affect health. Subsequently, more recently several clinical trials evaluated the possibility of distinct dietary patterns and nutrients to act as anti-ceramide regimes in humans. With nutrition being an important component of AIRD-related complications, the present review details the evidence regarding ceramide levels in patients with AIRDs, the results of anti-ceramide treatments and discusses the possibility of using medical nutritional therapy as a complementary anti-ceramide treatment in rheumatic disease.

## 1. Introduction

Autoimmune rheumatic diseases (AIRDs) are a set of diseases that include a wide spectrum of disorders and dysfunctions of the connective tissue, with the predominant involvement of the musculoskeletal system [1]. Common AIRDs include rheumatoid arthritis (RA), ankylosing spondylarthritis (SpA), systemic lupus erythematosus (SLE), fibromyalgia syndrome (FMS), primary Sjögren’s syndrome (pSS), systemic sclerosis (SSc), myositis, systemic vasculitis and psoriatic arthritis (PsA). All AIRDs present with related clinical manifestations, similar autoantibody responses and frequent transition from one entity to another [2]. Pain, fatigue, depression, disability and skin manifestations are frequent symptoms of these diseases [3], comprising a serious public health problem, as they induce a significant reduction in the physical activity levels of patients [4], are the main cause of disability including work disability [5], negatively impacting the quality of life of patients [6]. AIRDs carry a great disease burden globally, involving increasing medical costs [7], as well as major societal and indirect costs [8]. Subsequently, the economic impact of AIRDs on the health structures of patients with rheumatic conditions greatly exceeds that of other chronic diseases, such as cardiovascular disease (CVD) and cancer [9].

AIRD treatment is based on a strategic, comprehensive approach, with the primary aim being achieving and attaining disease remission, mostly on a treat-to-target (in many AIRDs) basis and maintaining immunosuppression, with the use of disease-modifying antirheumatic drugs (DMARDs) or biologics. Nonetheless, nutrition plays an important auxiliary role in the prevention and management of AIRDs [10]. A plethora of evidence has linked disease progression, severity, relapse and pharmacotherapy-induced adverse events to specific dietary interventions/changes [11,12,13,14,15,16,17]. Furthermore, the large majority of patients with AIRDs also demonstrate overweight and obesity, aggravated by physical inactivity [18,19]. In parallel, cachexia [20], micronutrient deficiencies [21,22,23,24,25], gut dysbiosis [26,27,28,29], poor diet quality [30,31,32] and malnutrition [33] are frequent nutritional issues among patients with rheumatic conditions; thus, the need for a comprehensive dietary approach and treatment is a necessity.

One of the important issues in the management of AIRDs involves the low target specificity which can induce negative, unpredicted effects on the level of cell metabolism [34]. As a result, many patients develop hyperlipidemias [35,36], and the majority exhibit an elevated cardiovascular (CV) risk [37,38], mainly as a result of dysregulated lipid metabolism [34]. To combat this undesirable issue, alternative metabolic pathways are being investigated, targeting inflammation while avoiding the metabolic side-effects of conventional anti-inflammatory treatments [34]. In this manner, anticeramide treatments have also been proposed as potential co-therapies in AIRDs [39,40] and a plethora of studies have examined factors associated with changes in ceramide concentrations among patients with an AIRD diagnosis.

## 2. Ceramides

### 2.1. General

Ceramides (Cers) are a large heterogeneous group of compounds, consisting of an 18-carbon amino alcohol, usually sphingosine and amide-linked aliphatic fatty acid chains of various sizes [41,42]. Due to the heterogeneity of ceramides in terms of (a) the number of carbon atoms in the fatty acids, (b) the location, (c) unsaturation and (d) hydroxylation, they are classified as a group of compounds and not as individual species [41,42]. They have various effects in humans, spanning from health promotion, to insulin resistance (IR) and atherogenesis-related effects. In addition, aside from sphingosine, there are ceramides biosynthesized with 6-H hydroxy-sphingosine, dihydro-sphingosine and phyto-sphingosine that also participate in ceramide generation, mainly of the skin [43]. In mammals, the number of carbon atoms in ceramides typically reaches up to 24 (although it can also be as high as 36), while unsaturation, the position and the degree of hydroxylation tend to vary among distinct ceramide species [44].

Ceramides are precursors of sphingolipids (glycosylceramides and sphingomyelins), a basic group of lipids that are the components of cell membranes. They also have an active role in various pathophysiological functions, cell communication and pathogen recognition [45]. In a similar role, ceramides acting as bioactive lipids also serve as signaling molecules for various processes including apoptosis (as pro-apoptotic molecules) [46], cell growth and differentiation, regulating the production of molecules such as sphingosine, the sphingosine 1-phosphate (S1P) and ceramide 1-phosphate (C1P) [47]. In general, they consist of signaling molecules altering the immune response [48] and driving endothelial dysfunction [49].

In a comprehensive review, Hannun and Obeid [45] summarized the majority of ceramides and other sphingolipids’ functions within the cell, as well as the types of molecules involved in each function. These include their key contribution to cell death and proliferation, participation in the aging process, autophagy, cytoskeleton rearrangement, mitophagy and cell migration [45]. Collectively, the evidence indicates that change in the concentration of various ceramides, due to endogenous factors (overexpression/inhibition of genes such as the ceramide synthases and sphingomyelinases) or exogenously, affects almost all the basic operations of the cell [45].

### 2.2. Ceramide Nomenclature

**Figure 1:**
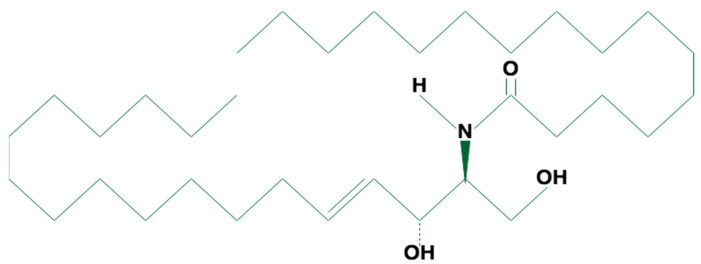
Structure of the C16 Ceramide (d18:1/16:0), created with ioDraw.

Ceramide characterization includes information about the number of carbon atoms and the unsaturation of the sphingoid base, the number of hydroxyls and the number of carbon atoms in the acyl chain [44]. The most common CVD risk-related ceramide molecules, the Cer(d18:1/16:0) (Figure 1), Cer(d18:1/18:0) and Cer(d18:1/24:1), compared with Cer(d18:1/24:0) are comprised of sphingosine, two hydroxyls (as for “d”), a double bond and a carbon acyl chain of 16, 18, 24 (and a double bond) and 24 carbon atoms, respectively [50]. Ceramides are also referred to using sphingosine, the number of carbon atoms and the double bonds of acyl chains only [51].

### 2.3. Metabolic Pathway of Ceramide Synthesis

Ceramides are generated through three distinct pathways. The first one involves de novo synthesis, in a four-step process beginning with the concentration of serine and palmitic acid. The first reaction is catalyzed by the enzyme serine palmitoyl-transferase (SPT), which is also one of the regulatory molecules of the synthesis pathway [41]. The remaining steps are catalyzed by the enzymes 3-ketosphinganine reductase, ceramide synthetase and dihydroceramide desaturase [42]. It appears that the de novo synthesis is activated either by the dietary accumulation of serine and/or palmitate, or in response to the intake of selected pharmaceutical agents, elevated stress or oxidized low-density lipoprotein (LDL) concentrations. Ceramides produced in this manner mediate the effects on stress and apoptosis [52].

The other two modes consist of bidirectional and recycling reactions of sphingolipids such as sphingomyelin (SM), glycosylceramides and more complex sphingolipids. In the second pathway, sphingomyelins are hydrolyzed to ceramides by sphingomyelinases, C1P by C1P phosphatases and glycosylceramides by acid β-glycosylceramidases [45,53]. Ceramides are produced in response to oxidative stress and tumor necrosis factor-alpha (TNF-α) [52]. The last way is the so-called rescue path. During this mode, complex sphingolipids are catabolized in late lysosomes and their sphingoid bases are converted to ceramides. It is used to regulate processes in the cell, such as apoptosis, growth arrest, cell signaling and molecule transport [52]. The enzymes that participate in ceramides’ synthesis pathways are situated in different places in the cell, with each pathway being activated independently. Thus, cells respond to the distinct stimuli they receive, depending on the pathway that is activated, as well as on where and at what time this takes place [52].

The activation of metabolic pathways occurs when lipid excess is apparent in the body. The tiles are used to manage this excess, so that the structure of the membranes is not modified. Specifically, tiles facilitate their conversion to acyl-CoA, activate the genes involved in lipid storage and increase the utilization of fat as an energy source [54]. Therefore, ceramides, in the same way as sphingolipids, participate in the regulation of metabolic pathways, where some of them lead to IR [55].

As for skin ceramides [56], after being synthetized in the endoplasmic reticulum, they enter the Golgi and are converted into glucosylceramides or SM. Then, they are transported to the stratum corneum through secretory vesicles [57]. In addition to their structural role in the epidermis, they also act as signaling molecules, regulating important functions such as apoptosis, proliferation (inhibition) and differentiation, with ceramides enhancing these processes, while S1P reverses them, acting as a growth factor [57,58].

### 2.4. Ceramides and Sphingolipids as Activators of Inflammation

Among their multiple important functions, lipids also serve as signaling molecules, cross-regulating inflammation [59,60]. For instance, it is known that the production of cytokines and chemokines is preceded by eicosanoids, whereas phosphoinositides also serve as lipid messengers [60,61]. More recently, many studies, mostly in vitro, have implicated ceramides and sphingolipids in general, in inflammation. Many of them concern the ceramides produced as a result of sphingomyelinase (SMase) actions [62]. TNF-α activates sphingomyelinases, which, in turn, promote the nuclear factor kappa-light-chain-enhancer of activated B cells (NF-κB) [63]. NF-κB, as a transcription factor, promotes the activation of several inflammatory cytokine genes, including interleukin-1β (IL-1β), interleukin-6 (IL-6), interleukin-8 (IL-8) and pro-inflammatory enzymes, including monocyte chemoattractant protein-1 (MCP-1) and cyclo-oxygenase 2 (COX-2) [64,65], the latter then promoting the upregulation of prostaglandin E2 (PGE2).

Other studies have revealed that ceramide synthesis alone can promote the synthesis of pro-inflammatory cytokines (IL-1β) and other inflammatory factors, such as TNF-α, interferon-γ (IF-γ) and platelet-activating factor (PAF) [66]. Conversely, these inflammatory factors (TNF-α, IF-γ, IL-1β, PAF) can further promote ceramide synthesis through the activation of sphingomyelinases, thus exacerbating inflammation [67].

The production of C1P via ceramide kinase, as well as lactosylceramide (LactCer), has been shown to activate signaling molecules, including the cytosolic phospholipase A2. This, in turn, promotes the production of arachidonic acid, thus inducing the synthesis of pro-inflammatory factors, such as leukotrienes and prostaglandins [68,69].

Regarding inflammation, some ceramides, including the d18:1/24:2 (N-stearoyl-D-erythro-sphingosine), d18:1/24:0 and S1P, have been found to increase during their duration and are characterized as pro-inflammatory. S1P, in particular, appears to regulate both the immune response and inflammation [70,71]. It binds to specific cell receptors participating in cell growth, angiogenesis, proliferation, lymphocyte migration and inhibition of apoptosis [72,73]. Today, both the S1P and the S1P receptor (S1PR) are considered as novel and interesting therapeutic targets for AIRDs [72]. In contrast, other ceramides, such as dihydroceramides and sphinganine, are considered as anti-inflammatory since they act in reducing inflammation [74].

## 3. Evidence Regarding Ceramides in Patients with AIRDs

### 3.1. Ceramides in Rheumatoid Arthritis (RA)

Rheumatoid arthritis (RA) is a degenerative, inflammatory arthritis, predominantly affecting the synovial joints, which gradually leads to the development of deformities and disability [75]. RA can also involve extra-articular manifestations in the muscles, skin, heart, lungs and other organs/tissues [76].

The possible mechanisms implicating ceramides in RA are described in earlier studies taking place both in vitro and in vivo (mainly in mouse models). These reveal that the Cer C2 (d18:1/2:0) induces apoptosis of rheumatoid synovial cells, through the modulation of various signaling pathways, including the inhibition of anti-apoptotic signals and the stimulation of pro-apoptotic ones [77]. In addition, experiments in mice showed that ceramides induce apoptosis of fibroblast-like synovial cells, thereby stopping their proliferation [78]. Ceramides are also involved in the signaling of IL-1β and TNF-α as secondary lipid messengers, but also in the synovial cells of patients with RA, causing apoptosis [39], all in a time-of-day dependent manner [79]. Studies investigating the concentration of ceramides in patients with RA are using samples primarily from the blood serum or the synovial fluid (SF) (Table 1).

Using serum samples, Miltenberger-Miltenyi et al. [84] sought to understand whether sphingolipids may serve as biomarkers in RA. The results revealed higher levels of monohexosylceramide (MHC), sphingosine (So) and ceramides in established RA compared with healthy controls, even after controlling for participants’ age and gender [84]. Interestingly, the MHC levels remained increased even after additionally controlling for medication use. The most significant of these biomarkers involved So, followed by MHC. On the other hand, patients with early RA also exhibited greater So concentrations compared to healthy controls [84].

These findings were further verified by other researchers. In a Taiwanese study [81], higher levels of S1P expression were found in SF samples of patients with RA compared to patients with osteoarthritis (OA) [81]. Similarly, greater sphingomyelinase activity was observed in the serum of patients with RA compared to healthy controls [80]. In a more recent study, greater serum LactCer levels were observed in patients with RA compared to controls [86]. LactCer activates the reactive oxygen species (ROS) and cytosolic phospholipase A_2_ (cPLA_2_a), releasing arachidonic acid, a known inflammation mediator [68].

A study of patients with RA compared the lipid profile in preclinical, active and prolonged remission [59], with the aim of investigating the metabolic pathways involved in the initiation, perpetuation and resolution of RA. Those with active RA, who were good responders to DMARD treatment, demonstrated higher serum ceramide 42:1 and SM, six months post-treatment. Moreover, a large difference was observed in the number of lipids differentially expressed in the serum and SF of patients with active RA, involving 15 and 135 lipids, respectively [59]. Moreover, Koh and associates [59] also observed severe perturbation in the lipidome profile in RA joint fluid, which was correlated with the extent of inflammation and severity of synovitis on ultrasonography, though not observed in the serum of patients with active RA. Thus, it seems that the overexpression of ceramides and SM is probably involved in the joint pathology of RA, but this is not always seen in the systemic circulation, with contradictory expression of lipids between the serum and SF being reported by Koh et al. [59]. Therefore, it appears that lipid metabolism is differentiated between joints and the blood stream, although a certain degree of communication between them might also exist [59].

In a lipidomic analysis of SF samples [82], elevated concentrations of SMs and ceramides were closely related to disease progression. The results also showed that ceramides constituted the second most important group of sphingolipids in the SF of patients with RA [82]. Six distinct species were recognized, with d18:0/24:0 being the most dominant (some of the remaining species were d18:1/16:0, d18:1/22:0, d18:1/23:0 and d18:1/24:1). In fact, their concentration was increased by 3.5 times (2.8–4.2-fold) in patients with RA compared to healthy controls. In addition, it was suggested that about 70% of them contained mainly saturated fatty acids (SFA). Hexosylceramide (HexCer) and dihexosylceramide (Hex_2_Cer) were detected, five species of each group. Compared to the control population, concentrations of HexCer species were increased by 5.8-fold, while Hex_2_Cer species were elevated by 6.9-fold in the RA population. SM species, and especially SM 34:1, were elevated by over 3 times in RA than in controls. In particular, a total of 19 SM species were 3 ½ times elevated in patients with RA, compared to the controls. Furthermore, the concentration of lipids was unrelated to the age of the individuals included in the study. These findings suggest that different ceramide species may be involved in the progression of RA. However, further research is required to demonstrate the role of extracellular species in synovial joints [59], as well as whether they are synthesized de novo or as the residue of overactivity of the sphingomyelinase enzyme [82].

Finally, Poolman and associates [85] added another dimension to the role of ceramides in RA, by showing that serum ceramides attained circadian rhythmicity, peaking at 23:00 h, suggesting that they are products of a newly rhythmic enzymatic pathway.

#### Ceramides as Therapeutic Targets in RA

Medcalf and associates [83] showed that 16 weeks of methotrexate (MTX) treatment normalized plasma metabolites, including ceramide levels. Research using animal models has shown more promising results. Yang [40] showed that glucocerebrosidase (GBA) administration improved arthritis in mice, and in parallel, circulating inflammatory mediators, including IL-1β, IL-6, IL-18 and matrix metalloproteinase (MMP)-1 levels were also improved. Similarly, pharmacological inhibition or genetic ablation of acid sphingomyelinase reduced symptoms of arthritis (including joint swelling) and circulating pro-inflammatory cytokines in the arthritic joints of mice [87]. Animal studies also indicate that the regulation of synovitis is greatly regulated through the S1P pathway [88]. In collagen-induced arthritis mice, the S1P receptor is upregulated in the synovial tissue and inflammation increases S1P/S1P3 signaling, which stimulates the overproduction of IL-6 in fibroblast-like synoviocytes [89]. Furthermore, rat models of RA suggest that proangiogenic factors stimulate the Sphk1/S1P/S1P_1_ pathway, upregulating proliferation and migration, while improving angiogenesis [90].

In parallel, in vitro studies revealed that the administration of C2 Cer in the synovial cells of patients with RA induced acute reversible morphological changes, as well as irreversible nuclear alterations, characteristic of apoptosis [39]. This ceramide-related apoptosis has also been noted in RA through the inhibition of anti-apoptotic kinases, such as the Akt, or the extracellular signal-regulated kinase ½ cascade (ERK1/2) [77]. Collectively, the data indicate that S1P could be a promising therapeutic target in RA, although more research is deemed necessary [88].

### 3.2. Ceramides in Ankylosing Spondylitis (SpA)

Ankylosing spondylitis (axial spondylarthritis) is a type of arthritis with a chronic and inflammatory form. The regions of the body most commonly predisposed are the spine and pelvis. The clinical pathology includes excessive proliferation of osteocytes in the areas of inflammation, resulting in ankylosis of the affected joints [91]. 

In a study comparing patients with RA, healthy controls and individuals with an SpA diagnosis [84] (Table 1), the latter group exhibited decreased levels of ceramides compared with the participants in the control arm.

#### Ceramides as Therapeutic Targets in SpA

El Jamal and colleagues [92] used mouse primary osteoblasts, chondrocytes and tenocytes as cell culture models, to assess S1P secretion and the expression of the sphingosine kinase 1 (Sphk1) gene. In SpA enthesis S1P was overproduced, as a residue of the elevated cytokines and mechanical stress. Furthermore, S1P might favor the abnormal ossification of the enthesis locally; thus, blockage of the S1P metabolic pathway was suggested as a novel, interesting therapeutic approach for treating SpA [92].

### 3.3. Ceramides in Systemic Lupus Erythematosus (SLE)

Systemic lupus erythematosus (SLE) is a chronic autoimmune disease with multisystem involvement [93]. Constitutional symptoms include fatigue, fever, muscle and joint pain, arthritis and body weight changes (weight loss, or sometimes weight gain) [93,94,95], but disease progression can lead to the total destruction of certain organs, including the lungs and kidneys. The diagnosis is usually formed between the second and fifth decade of life, yet the etiology of SLE remains unknown. Nonetheless, SLE progression is understood to be influenced by various environmental, hormonal and genetic factors, all interacting together [96]. A typical characteristic of the disease is the profoundly altered lipid metabolism. The “lupus pattern” is the SLE dyslipoproteinemia, which may evolve to the development of premature atherosclerosis and increased age-specific incidence of CVD [97,98]. Compared to healthy controls, patients with SLE exhibit a profoundly greater atherosclerosis risk, even when sharring similar CVD risk factors, including the Framingham score [99]. As a result, the role of ceramides has been examined in SLE (Table 2).

In an early study, Checa and associates [100] demonstrated that sphingolipids are, in fact, dysregulated in SLE and that this dysregulation is closely related to disease activity. In their study, the C16:0Cer was elevated in patients with SLE compared to healthy participants, but circulating levels were normalized after treatment with immune suppressants. Even within distinct SLE antibody subgroups though, the differences were apparent. Idborg [103] stratified patients with SLE according to their autoantibody profile in two subgroups, those with Sjögren’s syndrome-like SLE (the SSA/SSB+ group, where they were all positive for all three of SSA and SSB antibodies, though negative in the lupus anticoagulant (LA)) and those with antiphospholipid syndrome-like SLE (the aPL+ group, all positive in the LA test, but negative for all three of SSA and SSB antibodies). They found that the circulating levels of ceramide synthase 5 (CERS5), integrin subunit beta 1 (ITGB1) and solute carrier family 13 member 3 (SLC13A3) were higher among the SSA/SSB+ group, indicating that these patients might, in fact, have a greater benefit from interferon (IFN)-blocking therapies.

Similarly, Li and associates [105] showed that specific ceramide species, including the Cer (NS, n-acylated sphingolipid) (d18:1/18:0), Cer (NDS) (d18:0/16:0) and the Cer (NS) (d18:2/24:2) were observed in greater concentrations in patients with SLE. McDonald and associates [106] showed that in SLE, an altered profile of lipid raft-associated glycosphingolipids (GSLs) is apparent. In more detail, LactCer, monosialotetrahexosylganglioside (GM1) and globotriaosylceramide (Gb3) are increased in SLE, and all of these are associated with a greater liver X receptor β (LXRβ) expression. The LXRβ consists of a nuclear receptor controlling cellular lipid metabolism, while influencing acquired immune responses [106]. Other studies suggested that the altered lipid species—including sphingolipids—in SLE could predict interleukin-10 (IL-10) concentrations [97] and disease activity [102]. Moreover, disease activity was also related to the 18:0/18:2 diacyl phosphatidylethanolamine (dPE), explaining 22.6% of the observed variability in the SLE disease activity index (SLEDAI) [97].

A plethora of studies have suggested that sphingolipids are important effectors of renal function [108]. Using mice, Nowling [109] demonstrated that mice with lupus nephritis (LN) exhibited elevated LactCer and HexCer levels, as a possible result of greater ganglioside GM3 catabolism. The glomerular filtration barrier function greatly depends on maintaining the integrity of lipid rafts, which include sphingolipids [110]. With this in mind, Patyna [107] compared patients with LN and patients with SLE, free of renal injuries. The results revealed that chain-length specific ceramides in blood, most likely serum C24:1Cer concentrations, could act as potent biomarkers for renal impairment in patients suffering from SLE.

Given that African-Americans have a greater risk of developing SLE and accelerated CVD [111], Hamad and associates [101] investigated the effect of race on the sphingolipid profile of patients with SLE. The results revealed that in patients with SLE, the C16:0Cer/S1P ratio and the concentrations of C18:1 and C26:1 LactCer, C20:0 HexCer and sphingoid bases, may well be dependent on race.

#### Ceramides as Diagnostic Targets of CV Risk and Therapeutic Targets in SLE

According to Harden [112], the identification of S1P may prove an accurate prognostic factor for CV risk in SLE. S1P can stimulate the release of a plethora of inflammatory mediators including cyclooxygenase, the TNF-α and prostaglandins in the macrophages [113]. On the other end of the spectrum, targeted treatment reducing the formation of S1P or blocking its receptors might prevent the development of atherosclerosis in SLE [112]. Today, sphingolipid tests still lack the necessary specificity and sensitivity to be used alone as diagnostic tools in SLE [112].

Regarding the effect of standard treatment on the concentration of altered ceramide species, differences were noted between studies. Patyna and associates [107] failed to depict differences in the serum C24:1 levels of patients with SLE post-glucocorticoid (GC) treatment. On the other hand, Checa [100] noted that immunosuppressive therapy normalized all dysregulated sphingolipids in SLE. In line with these findings, Idborg and associates [104] showed that Rituximab treatment reduced the concentrations of all sphingolipids and in particular the dihydroceramide C16:0 and glucosylceramide C16:0 groups. Unfortunately, the use of ceramide-targeted treatments in humans is still limited, however, recently, the efficacy of cenerimod, an S1PR1 modulator [114], was evaluated [115]. Hermann and associates [116] applied a double-blind RCT design to evaluate the efficacy of cenerimod in SLE compared to placebo and showed that administration of 4 mg of cenerimod improved the modified SLEDAI-2000 (mSLEDAI-2K) and the anti-dsDNA antibodies, suggesting a clinical and biological improvement. McDonald and associates [106] used N-butyldeoxynojirimycin, a clinically approved inhibitor, to normalize GSL metabolism, correct CD4+ T cell signaling and functional defects and lower the production of anti-dsDNA antibodies by autologous B cells in patients with SLE, in vitro.

On the other hand, animal studies using SLE models showed that modulators of the S1PR (such as Fingolimod) can inhibit the migration of lymphocytes from lymphoid organs [117], offering a new therapeutic target for SLE. In parallel, the FTY720 was also used in animal models of SLE. The FTY720 consists of a novel immunosuppressant, greatly resembling sphingosine structurally, while targeting the S1PR. The results revealed a high degree of CD4-negative/CD8-negative T cell apoptosis (>70%), a reduction in anti-double stranded DNA (anti-ds-DNA) antibodies as well as in the deposition of IgG in the kidneys, and a prolonged survival in FTY720-treated mice, compared to the controls [118]. Finally, the Ozanimod (RPC1063), was also evaluated in the lupus mouse model for treating LN. Ozaminod is a modulator of S1PRs 1 and 5 and the treatment of mice in a 3.0 mg/kg dose induced a reduction in proteinuria, endocapillary proliferation, mesangial expansion, tubular atrophy, glomerular deposits, interstitial infiltrates and fibrosis [119]. Collectively, the findings suggest that sphingolipid and sphingolipid metabolism-targeted therapy appears promising for the prevention of disease progression in SLE [112].

### 3.4. Ceramides in Patients with Fibromyalgia Syndrome (FMS)

Fibromyalgia syndrome (FMS) is a chronic syndrome, with intense pain in the muscles and bones. FMS is related with the improper processing of pain, due to the incorrect functioning of the neurotransmission pathway [120]. It is also associated with other symptoms, including fatigue, cognitive decline and impaired sleep.

Table 3 details the case-control studies assessing the levels of ceramides in patients with FMS.

Using plasma samples, Caboni and associates [121] revealed that phosphocholine and ceramide lipids dominated the metabolite profile of patients with FMS compared to healthy controls. Hsu [122] additionally allocated patients with FMS to two phenotype subgroups, namely the pain-dominant subgroup (PG) or the soreness and pain subgroup (sng-dominant group (SG)). In comparison to healthy controls, concentrations of SM (d18:1/18:0) and C18:1 Cer/C22:1 Cer were higher in SG, but not PG patients [122], indicating that different biomarkers may discriminate sng and pain phenotypes in FMS and serve as novel therapeutic targets.

#### Ceramides as Therapeutic Targets in FMS

Using a newly developed mouse model, Hung and colleagues [123] showed upregulation of several lipids, including lysophosphatidylcholines (LPCs), phosphatidylcholines (PCs), SMs and ceramides, in stressed mice. Darapladib and antioxidants were effective in inhibiting LPC16:0 synthesis and alleviating the stress-induced hyperalgesia [123]. No other studies have been conducted evaluating the efficacy of the ceramide pathway in FMS treatment.

### 3.5. Ceramides in Patients with Psoriatic Arthritis (PsA)

Psoriatic arthritis (PsA) is a systemic, immune-mediated inflammatory arthropathy, associated with psoriasis. It appears that the underlying systemic inflammation propels an increased accumulation of pro-inflammatory oxidized lipids and an excessive lipid oxidation [124]. Studies have associated specific pro- and anti-inflammatory serum eicosanoids with the joint disease score [125]. As a result of this disturbed lipid metabolism, patients with PsA exhibit greater CVD and metabolic disorders’ risk.

The composition of the stratum corneum involves differentiated keratinocytes and extracellular lipids, including ceramides, cholesterol and free fatty acids (FA) [57]. As a result, research on ceramides in psoriasis plaques (PsO) is soaring [126,127,128,129], whereas studies assessing the concentration of ceramides in patients with PsA remain limited. Overall, research (Table 4) has showed that lower levels of ceramides are associated with a variety of skin conditions involving skin dryness and barrier disruption, including PsO [128,130]. Compared to healthy controls, patients with PsO demonstrate lower total serum concentration of ceramides and higher S1P concentrations [131]. On the other hand, those with PsO and PsA exhibit higher total circulating ceramides [131]. It is believed that these low total ceramides and increased S1P serum concentrations may be the residue of altered epidermal metabolism and composition [131]. In a secondary analysis of the same Polish data [132], patients were additionally divided into two subgroups, according to their alanine aminotransferase (ALT) blood levels. The results revealed that in patients with PsO (including those with PsA), lignoceric ceramides was positively correlated with the concentrations of ALT, suggesting that disturbances of sphingolipid and FA may, in fact, act as triggers for the development of liver disease [132].

#### Ceramides as Therapeutic Targets in PsA

In the case of PsA, the research indicates that ceramides can be potential therapeutic targets. They can be used to develop drugs aimed at restoring sphingolipid metabolism in psoriasis [57], targeting S1P receptors for more severe cases or targeting enzymes involved in ceramide generation (and changes in ceramide species) [126].

Ponesimod consists of a selective, reversible, orally active modulator of the S1P receptor 1 (S1PR_1_) internalizing S1PR_1_, thus inducing the desensitization of T and B cells [57,133]. Furthermore, ponesimod limits the exit of lymphocytes from secondary lymphoid organs, thus regulating lymphocyte trafficking [57,133,134]. In a phase II, double-blind, randomized, placebo-controlled, parallel-arm trial, intervention with ponesimod induced a 75% reduction in the psoriasis area and severity index (PASI) score of patients compared to 13.4% for the placebo arm, within 16 weeks [133]. Overall, ponesimod seems to be a promising therapy for PsA, although more research is required [135].

### 3.6. Ceramides in Primary Sjögren’s Syndrome (pSS)

Primary Sjögren’s syndrome (pSS) is an autoimmune disease associated with damage of the exocrine glands as a result of lymphocytic infiltrates of mononuclear cells in the lachrymal and salivary glands [136,137]. Clinical symptoms of pSS may include dry eyes and oral cavity, but premature atherosclerosis and increased CV risk may also be apparent [138]. Table 5 describes the studies conducted on patients with pSS and healthy controls, assessing differences in the levels and composition of ceramides species.

Hla [141] identified S1P as a promoter of cell survival and proliferation, whereas, on the other hand, ceramides and sphingosine seem to act as inhibitors to cell proliferation, stimulating apoptosis. Sphingosine kinase (SK) regulates the fine balancing of these three molecules, by converting sphingosine to S1P through sphingosine phosphorylation. Sekiguchi [140] showed that the signaling of the S1P receptor 1 (S1P_1_) may in fact modulate autoimmune phenotypes in pSS, through the immune and epithelial cells. Moreover, another case-control study [139] revealed differences in the lipidomic profiles of tears and saliva in patients with pSS compared to healthy controls, involving 29/86 individual lipid species.

#### Ceramides as Therapeutic Targets in pSS

Although research on humans is still limited, animal research appears more promising. In further detail, anti-ceramide treatment through myriocin for pSS decreased the expression of IFN-γ and Th1 frequency and suppressed the infiltration of inflammation within the salivary glands, achieving the maintenance of salivary flow rate [142]. Similar findings were also observed in another study. Greater bone morphogenetic protein 6 (BMP6) expression was observed in the salivary glands of 54% of a cohort of patients with pSS, and this correlated with a low unstimulated whole saliva-flow rate [143]. Inhibition of BMP6 signaling in mice reduced phosphorylation of the SMAD1/5/8 in the submandibular glands, reducing inflammation while inducing the recovery of the salivary gland function [143].

### 3.7. Ceramides in Systemic Sclerosis (SSc)

Systemic sclerosis (SSc) is a chronic complex systemic autoimmune disease, targeting the connective tissue at the cellular level (fibroblasts and myofibroblasts) and the vasculature, while altering components of the innate and adaptive immunity [144]. SSc is characterized by a chronic, progressive ischemia and fibrosis of the organs and tissues, exhibiting great patient-to-patient variability, while leading to irreversible damage [145]. Several factors have been implicated in the development and progression of fibrosis, including vascular, epigenetic and immunologic pathways, all consisting of important targets for disease-modifying treatment approaches [145].

In this manner, the important role of ceramides in tissue fibrosis identified them as a promising target for the development of novel antifibrotic treatments [146,147]. In a case-control study of patients having a SSc diagnosis, Geroldinger-Simić and associates [148] revealed differences in the level of phospholipids (plasmalogens and SM) in the plasma of patients, compared to healthy controls (Table 6). Additionally, they demonstrated that distinct clinical manifestations of SSc are associated with different alterations in the metabolism of phospholipids. In a secondary analysis of the same study [149], the authors also identified four distinct dysfunctional metabolic pathways in SSc, namely the urea cycle, the kynurenine pathway, the gut microbiome and the metabolism of lipids. All four altered metabolic pathways were associated with dysregulated metabolites (including selected ceramides), vascular damage, inflammation, gut dysbiosis and fibrosis, and might be implicated in the pathophysiology of SSc [149].

#### Ceramides as Therapeutic Targets in SSc

Sphingosine and ceramides are important mediators in tissue fibrosis; thus, they both consist of a promising target for antifibrotic therapies [146,147]. In vitro studies [150] revealed that SSc fibroblasts exhibit greater resistance to Fas-mediated apoptosis and lower ASMase and transforming growth factor beta (TGFβ) expression, with the latter being an important fibrogenic cytokine in SSc. When ASMase is activated, it hydrolyzes sphingomyelin to ceramide, initiating the formation of ceramide rafts and the apoptotic process [151]. The sensitivity of the SSc fibroblasts to Fas-mediated apoptosis was restored by the forced expression of ASMase within the cells [150,152]. In SSc animal models, the S1P receptor 5 was shown to regulate fibrosis at the early stages of its development [153]. Furthermore, the S1P has a strong profibrotic action in SSc and controls fibroblasts, with dihydrosphingosine-1-phosphate (dhS1P) having a significant modulatory role in the regulation of phosphatase and tensin homolog levels [154]. Moreover, it was shown that the depletion of either the S1PR_1_ or the S1PR_2_ prevented the deleterious effects of S1P and dhS1P in fibroblasts in vitro [154], opening new avenues for the prevention of fibrosis in SSc.

### 3.8. Ceramides in Myositis

Idiopathic inflammatory myopathies is an umbrella term including dermatomyositis (DM), polymyositis (PM), inclusion body myositis (IBM), anti-synthetase syndrome and necrotizing myopathy (NM) [155,156]. They are characterized by chronic muscle inflammation leading to progressive muscle weakness. Table 7 presents the studies assessing ceramide levels in patients with myositises.

Dvergsten and associates [157] compared metabolite factors in the blood samples of patients with probable/definite juvenile DM (JDM) and healthy controls. Two of the identified factors were associated with JDM, including one acylcarnitine and one ceramide. JDM treatment induced a reduction in the concentration of both metabolites. To follow up this hypothesis, Lollel et al. [158] compared selected gene expression in the skeletal muscle biopsies of adult patients with DM or PM, pre and post-immunosuppressant treatment initiation, in a randomized controlled manner. The results revealed that the immunosuppressant regime altered the expression of genes involved in the metabolism of lipids, suggesting a potential lipotoxic treatment effect on the muscles.

#### Ceramides as Therapeutic Targets in Myositis

The effect of standard myositis therapy on the levels of ceramides has been examined in the literature. Sirolimus (rapamycin) consists of an inhibitor of the mammalian target of rapamycin (mTOR) pathway and an immunosuppressant drug used in myositis. Yamane and associates [159] showed that rapamycin promoted TGF-β signaling inducing an upgrade of the ceramide synthesis within the keratinocytes in vitro. Lollel and associates [158] showed that treatment with immunosuppressants enhanced genes that favor lipogenesis and lipid storage, such as the ceramide synthase 3 (CERS3), and induced a reduction in the expression of the Sphk1, suggesting enhanced accumulation of ceramides, implicated in lipotoxicity [160]. The authors concluded that immunosuppressive treatment appears to have a potential lipotoxic effect on muscles [160]. Nonetheless, it appears that myositis is as yet under-researched in terms of anticeramide treatments.

### 3.9. Ceramides in Systemic Vasculitis (SV)

Systemic vasculitis (SV) includes a wide spectrum of distinct diseases with variable clinical manifestations. Giant cell angeitides and giant cell arteritis, necrotizing angeitides, such as Kawasaki disease (KD), polyarteritis nodosa, Henoch–Schönlein purpura, Wegener’s granulomatosis or Churg–Strauss syndrome, all fall within the SV spectrum [161]. 

Anti-neutrophil cytoplasmic antibody (ANCA)-associated vasculitis (AAV) is a necrotizing vasculitis, with three distinct clinical types, namely the granulomatous one with polyangiitis (GPA), the eosinophilic granulomatous polyangiitis (EGPA) and the microscopic polyangiitis (MPA) [162]. Patients with AAV are in a hypercoagulable state [72,163], exhibiting more frequent thromboembolisms and cardiovascular and cerebrovascular events compared to healthy volunteers [164]. This is due to several factors, one of which involves the S1P. Higher plasma concentrations of S1P stimulate neutrophils and the production of thrombin, which can dismantle endothelial cells’ integrity during coagulation [72,165,166]. Hao and associates [70] compared the levels of S1P in patients with AAV, when in active disease and when in remission, with the sample comprising the cases and the control patients, at the same time (Table 8). The results showed that plasma S1P concentrations were higher in those with active disease. Moreover, the mean CD88 expression fluorescence intensity (MFI) value in S1P-triggered neutrophils was upregulated, indicating that S1P primes neutrophils for ANCA-related degranulation and respiratory burst [70]. S1PR antagonists reduced the production of oxygen radical species in C5a neutrophils due to ANCA-positive IgG, thus S1P inhibition appears to block the migration of C5a-primed neutrophils. These findings were also verified in a similar study [167]. In a case-control comparison, higher plasma S1P levels were observed in patients with active AAV compared to those in remission [167].

KD is an acute systemic vasculitis of the medium-sized vessels, affecting mostly infants and toddlers, and is the most common primary childhood vasculitis [170]. Comparison of acid sphingomyelinase (ASM) levels between patients with KD and controls revealed greater levels in the first compared to the latter, indicating the involvement of ASM in the pathophysiology of KD (Table 8) [168]. Although higher serum secretory ASM activity is considered as a consequence of the acute KD-induced inflammation, extracellular-secreted ASM appears to be a contributing factor in the development of KD and its prognosis [168]. Furthermore, serum ASM activity was also correlated to the concentrations of c-reactive protein (CRP), although no relationship was apparent with IL-6 levels [168].

Finally, IgA vasculitis (IgAV), also known as Henoch–Schönlein purpura, consists of a vasculitis of the small vessels, with a characteristic IgA1-dominant immune deposition at the walls of the vessels, presented either as a systemic or a single-organ-limited vasculitis [171,172]. In a case-control study, Liu [169] compared serum samples of patients with IgAV and healthy controls and observed that a total of 31 lipid ions were altered in IgAV, all belonging to six distinct classes, namely ceramides, triacylglycerols (TGs), PEs, PCs, phosphatidylserine and LPCs.

#### Ceramides as Therapeutic Targets in SV

Animal studies using S1PR modulators in autoimmune vasculitis revealed that treatment with Fingolimod (FTY720) improved symptoms of proteinuria and hematuria, and reduced both the formation of glomerular crescent and pulmonary hemorrhage [173]. In parallel, a reduction in renal T-cell infiltration was noted, S1PR_1_ mRNA was upregulated in contrast to renal IL-1β levels which were downregulated, while ANCA concentrations remained unchanged [173]. Collectively, these findings suggest that the possible therapeutic effects of the FTY720 appear to be B-cell independent [173].

## 4. Dietary Interventions as Anti-ceramide Treatments

As most ceramides appear to be synthesized de novo, diet appears to be a major contributor to ceramide composition and the level of circulating ceramides in humans [174]. Fatty acids from the diet are expected to be reflected in the ceramide composition [175,176].

According to Mah [174], the majority of ceramide synthesis is diet-centric; thus, dietary interventions may change ceramide concentrations in the blood and subsequently influence health (Table 9) [177]. Several intervention trials have studied the effect of diet on ceramides and in relation to difference health outcomes. Since only one study was performed in patients with AIRDs [178], we discuss here the effect of diet in healthy, overweight and individuals with increased CVD risk.

### Dietary Interventions as Anti-ceramide Treatments, Delivered in Randomized Controlled Trials

The FRUVEDomic pilot study achieved a reduction in the circulating ceramides of participants, including the C24:0 Cer, which is a known inhibitor of insulin signaling [184]. The intervention involved a diet rich in fruit and vegetables but restricted in SFA and refined carbohydrates. Moreover, improved inflammatory status, as assessed through the circulating cytokine concentrations, was correlated with ceramide levels [184]. Similar interventions were also applied in the Framingham Offspring cohort [191] with participants being allocated to a Mediterranean diet (MD) or a lacto-ovo-vegetarian diet (VD). The results revealed that the MD was associated with lower C16:0 and C20:0 concentrations. In the PREvención con DIeta MEDiterránea (PREDIMED) RCT, participants with a high CVD risk adhered to a MD supplemented with nuts or extra-virgin olive oil (EVOO), or continued their habitual diet [188]. The results showed that in the two active interventions, a higher ceramide score did not coincide with a high CVD risk, however, in the control arm, an elevated ceramide score was associated with a significantly higher CVD risk [188]. Finally, an additional RCT revealed that adherence to a VD reduced the ceramide concentrations, compared to the standard medical nutrition therapy for patients with coronary artery disease [182].

As far as lipid replacement RCTs are concerned, an Italian RCT [186] also examined the effect of EVOO against palm oil in improving ceramide concentrations. The results revealed that partial replacement of SFAs with MUFAs in the form of a chocolate-spread integrated in a isocaloric diet reduced the detrimental effects of SFAs on insulin sensitivity and reduced circulating harmful sphingolipids in young adults [186].

The LIPOGAIN-2 trial [185] investigated the effect of muffins eaten on top of the habitual diet for 4 weeks, by individuals with overweight or obesity. Muffins were either rich in SFA or PUFA and the results revealed that SFA intake markedly induced liver fat and serum ceramides, whereas dietary PUFA prevented liver fat accumulation and reduced ceramides and hyperlipidemia during excessive energy intake and weight gain in individuals with overweight.

Airhart and associates [179] showed that medium-chain fatty acids (MCFAs) might have more health benefits for patients with type 2 diabetes mellitus compared with the long-chain fatty acids (LCFAs). In further detail, 14 days of adhering to a high-MCFA diet decreased a variety of plasma sphingolipids, ceramides and acylcarnitines implicated in diabetic cardiomyopathy, inducing changes in several sphingolipids correlated with improved fasting insulins.

The pivotal effect of dietary fat on the circulating ceramide levels is apparent even in short-term trials. For instance, Tuccinardi [187] showed that the consumption of two walnut smoothies within a period of five days induced a significant reduction in the circulating harmful ceramides, in particular HexCers and SMs, which are known effectors of CV risk. Similarly, a short-term (four days) intervention with a MD or a fast-food diet revealed that four days of dietary modification were adequate for inducing the remodeling of the HDL lipidome, with certain lipid classes being more sensitive markers of diet [190].

Le Barz and associates [183] provided three different cream cheeses in post-menopausal women, all with different polar lipid (PL)-enriched milk. The results revealed that 4 weeks of intervention decreased the serum atherogenic ceramides species and these reductions were positively correlated with the reduction in total cholesterol (TC), low-density lipoprotein (LDL) and apolipoprotein B (ApoB), resulting in an overall improvement of CV risk markers.

When routine intake of sugar-sweetened beverages was replaced by the consumption of reduced fat milk in young adolescent boys for three weeks, decreases in systolic blood pressure and glycosphingolipid concentrations were noted, suggesting an overall favorable effect on CV risk following a short-term dietary intervention [181].

In the only RCT using participants with an AIRD diagnosis, Lindqvist and associates [178] applied a dietary cross-over intervention to improve the serum lipid profile of patients with RA, towards a less atherogenic one. The Anti-Inflammatory Diet In Rheumatoid Arthritis (ADIRA) trial showed that, although several components of the ceramide-and phospholipid-based CVD risk score (CERT2) were improved post-intervention, no significant difference was observed in the CERT2 of the intervention-receiving arm, compared to the control diet [178]. Furthermore, the trial revealed that a “healthier” composition of cholesteryl esters, phosphatidylcholines, alkenylphosphatidylcholines, TG and alkylphosphatidylcholines was apparent after the MD intervention was conducted [178].

Last, but not least, oral nutrient supplementation (ONS) studies have also been conducted. When vitamin D_3_ was administered to subjects with overweight/obesity, an increase in serum *N*-stearoyl-sphingosine (d18:1/18:0) (C18Cer) and stearoyl sphingomyelin (d18:1/18:0) (C18SM) concentrations was observed 16 weeks post-supplementation, in a dose–response fashion [180]. On the other hand, when dietary anthocyanins were administered in patients with dyslipidemia for 12 weeks in total [189], a dose-dependent reduction was observed in the plasma concentrations of all six ceramide species.

## 5. Conclusions

The present review presented all available evidence indicating changes in the concentrations and/or type of ceramides between patients with AIRDs compared to control groups. Even among patients with the same diagnosis, the observed differences correspond to the form, or severity and progression of the disease [126]. On the other hand, ceramides are also associated with the development and presentation of a plethora of AIRD comorbidities, including dyslipidemia, increased CVD risk, fibrosis, etc. Research is still in an early stage of development, and the degree to which dietary improvement of ceramide concentrations also leads to a decrease in disease activity, reduced inflammation and lowered risk for CVD in patients with AIRDs remains an issue that needs to be further investigated.

Today, many of the existing therapies for AIRDs act on the lipid metabolic pathways in order to exert their therapeutic effects [34]. Undeniably, ceramides consist of a novel therapeutic opportunity that may help us reduce the number and severity of immuno-metabolic complications and halt the elevated CVD risk associated with AIRDs [34]. A better understanding of the ceramide function and S1P pathobiology is anticipated to improve the management of AIRDs [192].

## Figures and Tables

**Table 1:** Summary of studies assessing ceramide levels in patients with rheumatoid arthritis (RA).

First Author	Origin	Participants	Biofluids	Results
Hanaoka [80]	USA	Cases: *n* = 33 patients with RA Controls: *n* = 17 non-RA controls	Blood serum	S-Smas activity in patients with RA was 1.4 times higher than in controls.
Huang [81]	Taiwan	Cases: patients with RA Controls: patients with OA	SF	Greater levels of S1P expression in patients with RA compared to controls.
Koh [59]	UK	Cases: *n* = 42 patients with active RA, *n* = 19 patients with RA on remission Controls: *n* = 18 preclinical RA and *n* = 49 patients with OA	SF, blood serum	An increase in serum Cer 42:1 and SM was observed in RA. The number of lipids differentially expressed in the serum and SF of patients with active RA *vs*. OA differed (15 and 135 lipids, respectively).
Kosinska [82]	Germany	Cases: *n* = 9 deceased, *n* = 18 patients with RA Controls: *n* = 30 patients with OA	SF	Six species of ceramides were identified, with d18:0/24:0 being the predominant one (other species: d18:1/16:0, d18:1/22:0, d18:1/23:0 and d18:1/24:1). Their concentration was increased (thrice) compared to the levels observed in the control arm (OA). About 70% of these contained mainly SFA. In addition, SM species were increased (three-fold) in RA compared to the OA.
Medcalf [83]	USA	Cases: *n* = 20 patients with RA initiating MTX therapy (15 mg/week) before and after 16 weeks of treatment Controls: *n* = 20 healthy controls	Plasma	RA induces alterations to the plasma metabolome. MTX therapy can partially correct these alterations involving TG, FA and ceramides.
Miltenberger-Miltenyi [84]	Portugal	Cases: *n* = 19 patients with estRA, *n* = 18 untreated patients with earlRA Controls: *n* = 13 untreated patients with early arthritis, not fulfilling the criteria for RA (non-RA), *n* = 12 patients with SpA and *n* = 20 healthy controls	Serum samples	Patients with estRA exhibited greater concentrations of So, MHC and ceramides compared to controls, when controlling for age and gender. MHC levels remained increased even after additionally controlling for medication. On the contrary, patients with SpA exhibited significantly lower levels of ceramides, in both analyses.
Poolman [85]	UK	Cases: *n* = 10 adults with a clinical diagnosis of RA (seropositive for RF and/or ACPA) Controls: *n* = 10 healthy, age- and gender-matched controls	Serum samples	No effect was observed in ceramides by serum lipids, and there was no effect of age, sex or BMI. More rhythmic lipids were apparent in the RA arm, particularly ceramides. Peak times for the newly rhythmic ceramides occurred at 23:00, indicating that they were products of a newly rhythmic enzymatic pathway.
Smesam [86]	Iraq	Cases: *n* = 118 patients with RA Controls: *n* = 50 age- and sex-matched healthy controls	Blood serum	LactCer levels were significantly elevated in patients with RA compared to controls.

ACPA, anti-citrullinated peptide antibodies; ADA, Adenosine deaminase; BMI, body mass index; Cer, ceramide; estRA, established RA; earlRA, early RA; FA, fatty acids; LactCer, Lactosylceramide; MHC, monohexosylceramide; MTX, methotrexate; RA, rheumatoid arthritis; RF, rheumatoid factor; S1P, sphingosine 1-phosphate; SF, synovial fluid; SFA, saturated fatty acids; SM, sphingomyelin; So, sphingosine; SpA, Ankylosing Spondylitis; S-Smas, secretory sphingomyelinase; TG, triglycerides; UK, United Kingdom; OA, osteoarthritis.

**Table 2:** Summary of studies assessing ceramide levels in patients with SLE.

First Author	Origin	Participants	Biofluid	Results
Checa [100]	Sweden	Cases: *n* = 107 female patients with SLE Controls: *n* = 23 healthy participants	Plasma	Higher levels of sphingolipids (Ceramides and HexCer) and lower levels of sphingoid bases were observed in SLE compared to controls. The ratio of C16:0Cer/S1P was the best discriminator between patients and controls, associated with disease activity but not with accumulated damage (SDI). Levels of C16:0- and C24:1-HexCers were able to discriminate patients with current *vs*. inactive/no renal involvement. Dysregulated sphingolipids were normalized post-immunosuppressive treatment.
Hamad [101]	USA	Cases: *n* = 73 patients with SLE Controls: *n* = 34 healthy controls (negative for autoimmune disease by the CSQ)	Plasma	In SLE greater levels of ceramides, sphingoid bases and their phosphates were observed compared to controls. A-A with SLE had higher levels of ceramides, HexCers, sphingosine and DH-S1P compared to healthy A-A. Caucasians with SLE exhibited greater levels of sphingoid bases, but lower ratios of C16:0Cer/S1P and C24:1Cer/S1P compared to same-race healthy controls. Caucasians with SLE + atherosclerosis had lower levels of sphingoid bases compared to atherosclerosis-free Caucasians with SLE. In contrast, A-A with SLE + atherosclerosis had greater levels of sphingoid bases and SMs compared to their same-race atherosclerosis-free peers. Compared to Caucasians with SLE + atherosclerosis, A-A patients had higher levels of selected sphingolipids. Plasma levels of sphingosine, C16:0Cer/S1P ratio and C24:1Cer/S1P ratio correlated with the SLEDAI among A-A.
Huang [102]	China	Cases: *n* = 40 patients with SLE of different clinical activity (including low and high activity) Controls: *n* = 10 healthy individuals	Blood	An imbalanced lipid metabolism, in particular sphingolipids, accompanied by dysregulated levels of apolipoproteins was observed, contributing to the disease activity of SLE.
Idborg [103]	Sweden	Cases: *n* = 378 well-characterized patients with SLE grouped by autoantibody profiling (APS-like SLE (aPL+) and SS-like SLE (SSA/SSB+)) Controls: *n* = 316 individually matched population controls	Plasma	In the SSA/SSB+ subgroup, the CERS5, RF and IgG were all increased.
Idborg [104]	Sweden	Cases: *n* = 10 patients with SLE (before–after study)	Plasma	Rituximab treatment downregulated sphingolipid levels. Differences between before and after treatment levels were observed for dihydroceramide C16:0 and glucosylceramide C16:0, as well as for 7 additional sphingolipids.
Li [105]	China	Cases: *n* = 17 patients with SLE Controls: *n* = 17 healthy participants	Serum	Specific ceramides, including Cer (NDS) (d18:0/16:0), Cer (NS) (d18:1/18:0) and Cer (NS) (d18:2/24:2) were higher in SLE. The ROC showed that the AUCs of these differential features were > 0.75, with Cer (NDS) (d18:0/16:0) reaching 0.958, and FA 20:4 reaching 0.875.
Lu [97]	China	Cases: *n* = 30 female patients with SLE Controls: *n* = 30 healthy individuals	Serum	The composition of specific lipid species including dPE (16:0/18:2, 16:0/22:6, 18:0/18:2, 18:0/22:6, 18:0/20:4), 18:2 LPC and Cer (N22:0 and N24:1) were altered in patients with SLE. All altered lipid species could predict IL-10 concentrations. The SLEDAI correlated to the 18:0/18:2 dPE, explaining 22.6% in the SLEDAI variability.
McDonald [106]	UK	Cases: *n* = 58 patients with SLE Controls: *n* = 36 healthy donors	Plasma	Compared with healthy controls, CD4+ T cells from patients with SLE displayed an altered lipid profile, in particular, of GSL with LactCer, Gb3 and GM1 levels being increased. Higher GSL in SLE were associated with greater LXRβ expression. Inhibition of GSL biosynthesis in vitro using N-butyldeoxynojirimycin, normalized GSL metabolism, corrected CD4+ T cell functional defects and signaling, and reduced the production of anti-dsDNA antibody by autologous B cells in patients with SLE.
Patyna [107]	Germany	Cases: *n* = 17 patients with SLE, free of renal injuries (eGFR ≥ 80 mL/min/1.73 m^2^ and alb/cr ≤ 30 mg/g) and *n* = 29 patients with LN (eGFR < 80 mL/min/1.73 m^2^ and alb/cr > 30 mg/g) Controls: *n* = 36 healthy individuals	Plasma and serum	Concentrations of specific ceramides (C16Cer, C18Cer, C24:1Cer, C20Cer) were higher in patients with biopsy-proven LN compared to SLE without renal injuries and controls. C24:1dhCer levels were elevated (plasma and serum) in LN compared to patients with SLE. Sphingosine levels were elevated (plasma and serum) in LN patients compared to controls. Sphinganine levels were elevated in LN compared to controls and to patients with SLE. Greater plasma S1P and SA1P levels were observed in patients with SLE and LN compared to controls. ROC analyses of the most altered species (C16Cer, C18Cer, C20Cer, C24:1Cer) between LN and SLE had a high diagnostic differentiation. GC treatment did not affect serum C24:1Cer levels.

A-A, African-Americans; Alb, albumin; anti-dsDNA, anti-double stranded DNA; APS, antiphospholipid syndrome; AUC, area under the curve; Cer, ceramide; CERS5, ceramide synthase 5; Cr, creatinine; CSQ, Connective Tissue Disease Screening Questionnaire; DH-S1P, Dihydro-sphingosine-1-phosphate; dPE, diacyl phosphatidylethanolamine; eGFR, estimated glomerular filtration rate; FA, fatty acid; Gb3, globotriaosylceramide; GC, glucocorticoid; GM1, monosialotetrahexosylganglioside; GSL, lipid raft-associated glycosphingolipids; HexCer, hexosylceramide; IgG, immunoglobulin G; IL-10, interleukin-10; LN, lupus nephritis; LPC, Lysophosphatidylcholines; LXRβ, liver X receptor β; NS, n-acylated sphingolipid; RF, rheumatoid factor; ROC, receiver operating characteristic; S1P, sphingosine-1-phosphate; SA1P, sphinganine-1-phosphate; SDI, Systemic Lupus International Collaborating Clinics/American College of Rheumatology Damage Index; SLE, systemic lupus erythematosus; SLEDAI, Systemic Lupus Erythematosus Disease Activity Index; SM, sphingomyelins; SSA, antibodies to Ro; SSB, antibodies to La.

**Table 3:** Summary of studies assessing ceramide levels in patients with FMS.

First Author	Origin	Participants	Biofluid	Results
Caboni [121]	Italy	Cases: *n* = 22 patients with persistent FMS Controls: *n* = 21 healthy controls	Plasma samples	Phosphocholine and ceramide lipids dominated the metabolite profile of patients with FMS.
Hsu [122]	Taiwan	Cases: *n* = 30 patients with FMS Controls: *n* = 25 healthy controls	Urine and serum samples	Potential FMS-relevant biosignatures included serum SM(d18:1/18:0). Higher levels of SM(d18:1/18:0) were observed in FMS compared to controls. Moreover, concentrations of C18:1 Cer/C22:1 Cer were also higher in SG, but not PG patients, compared to healthy controls.

Cer, ceramide; FMS, fibromyalgia syndrome; PG, pain-dominant group; S1P, sphingosine-1-phosphate; SG, sng-dominant group; SM, sphingomyelin.

**Table 4:** Summary of case-control studies assessing ceramide levels in patients with PsA.

First Author	Origin	Participants	Biofluids	Results
Myśliwiec [131,132]	Poland	Cases: *n* = 85 patients with exacerbated PsO (14 of which also had PsA) Controls: *n* = 32 sex- and age-matched healthy controls	Serum ceramides and S1P	Total serum concentration of ceramides was decreased and S1P levels were increased in patients with PsO compared to controls. Among those with PsO, no correlations were established with disease activity and inflammation markers. Only those with PsA exhibited greater total ceramide concentrations. Serum sphingolipid disturbances were also observed in PsO. Lowered total ceramides and increased S1P serum levels may reflect the altered epidermal metabolism and composition. In PsA greater total ceramide concentrations was observed than in PsO involving the skin only.

Cer, ceramide; PsA, psoriatic arthritis; PsO, psoriasis; S1P, sphingosine-1-phosphate.

**Table 5:** Summary of case-control studies assessing ceramide levels in patients with pSS.

First Author	Origin	Participants	Biofluids	Results
Fineide [139]	Norway	Cases: *n* = 10 female patients with pSS Controls: *n* = 10 age- and gender-matched healthy participants	Saliva and tear samples	Differences were observed in the lipidomic profiles of saliva and tears in patients with pSS compared to controls. Differences in 29/86 individual lipid species were also noted in stimulated saliva, and these were comparable to the glandular biopsies. In pSS, an increase in SM and dPC and a decrease in DAG and ceramides was noted, compared to controls.
Sekiguchi [140]	Japan	Cases: *n* = 5 patients with pSS Controls: *n* = 6 healthy volunteers	Peripheral blood and LSP biopsy	In advanced stages of pSS, the expression of S1PR_1_ was enhanced in inflammatory mononuclear cells. S1P enhanced the proliferation and IFN-γ production by CD4+ T cells. Compared to healthy controls, in patients with pSS the enhancing effect of S1P on IFN-γ production by CD4+ T cells was stronger in the latter. Additionally, S1P enhanced the expression of Fas and Fas-mediated caspase-3 induction in epithelial cells of the salivary gland.

DAG, diacylglycerol; dPC, diacylglycerophosphocholine; IFN-γ, interferon-γ; LSP, liver membrane lipoprotein; pSS, primary Sjögren’s syndrome; S1P, sphingosine-1-phosphate; S1P_1_, Sphingosine-1-phosphate receptor 1; SM, sphingomyelin.

**Table 6:** Summary of studies assessing ceramide levels in patients with systemic sclerosis (SSc).

First Author	Origin	Participants	Biofluid	Results
Geroldinger-Simić [148,149]	Austria	Cases: *n* = 52 patients with SSc Controls: *n* = 48 controls without SSc	Plasma	Significant differences were observed in the level of phospholipids (plasmalogens and SMs) in patients with SSc compared to controls. PC plasmalogens species and SM were greater in SSc plasma compared to healthy plasma. Moreover, a significant association was noted in the metabolism of phospholipids (PC and PE plasmalogens species and SM) with different clinical manifestations of SSc.

PC, phosphatidylcholine; PE, phosphatidylethanolamine; SM, sphingomyelin; SSc, systemic sclerosis.

**Table 7:** Summary of studies assessing ceramide levels in patients with myositis.

First Author	Origin	Participants	Biofluid	Results
Dvergsten [157]	USA	Cases: *n* = 10 patients with probable (*n* = 4) or definite (*n* = 6) JDM Controls: *n* = 9 healthy controls	Blood samples	Of the 8 PCA–derived metabolite factors (one AC, two AA, three sphingosine and two ceramides), two were associated with JDM (one AC and one ceramide). All identified factors decreased with JDM treatment.
Lollel [158]	Sweden	Cases: *n* = 6 adult patients with DM/PM (before-after study)	Skeletal muscle biopsies	After treatment with immunosuppressants, the expression of genes involved in lipid metabolism was altered, suggesting a potential lipotoxic effect on muscles of the immunosuppressive treatment.

AA, amino acid; AC, acylcarnitines; Cer, ceramide; DM, dermatomyositis; JDM, juvenile dermatomyositis; PCA, principle component analysis; PM, polymyositis; SM, sphingomyelin.

**Table 8:** Summary of studies assessing ceramide levels in patients with vasculitis.

First Author	Origin	Participants	Biofluid	Results
Hao [70]	China	Cases: *n* = 29 patients with AAV (active/in remission)	Plasma samples	The circulating S1P was higher in patients with active AAV compared with patients in remission.
Konno [168]	Japan	Cases: *n* = 15 patients with acute KD (before/after IVIG-treatment study) Controls: *n* = 9 healthy participants and *n* = 4 children with adenovirus infection	Serum	Serum ASM activity before IVIG was elevated in patients with KD compared to controls, indicating the involvement of ASM in the pathophysiology of KD. Serum ASM activity before IVIG was correlated to the circulating CRP levels.
Liu [169]	China	Cases: *n* = 58 patients with IgAV Controls: *n* = 28 healthy controls	Serum	A total of 31 lipid ions were altered in IgAVs, belonging to six classes, namely, TG, PE, PC, phosphatidylserine, ceramides and LPC.
Sun [167]	China	Cases: *n* = 32 patients with active AAV Controls: *n* = 20 patients with AAV in remission	Plasma samples	S1P concentrations were greater in patients with active AAV compared to patients in remission. Plasma levels of S1P were correlated with the serum creatinine concentrations and inversely related to the eGFR. S1PR1–5 was expressed on glomeruli endothelial cells and S1PR1 4 and 5 were expressed on neutrophils.
Wu [72]	China	Cases: *n* = 40 patients with AAV Controls: *n* = 10 healthy controls	Plasma samples	In AAV, levels of S1P were related to the D-dimer, PLT and BVAS levels. Therefore, plasma S1P can be a biomarker predicting coagulation-related complications in AAV.

AAV, ANCA-associated vasculitis; ANCA, anti-neutrophil cytoplasmic antibody; ASM, Acid sphingomyelinase; BVAS, Birmingham vasculitis activity score; CRP, c-reactive protein; eGFR, estimated glomerular filtration rate; IgAV, IgA vasculitis; IVIG, intravenous immunoglobulin; KD, Kawasaki’s disease; LPC, lysophosphatidylcholine; PC, phosphatidylcholine; PE, phosphatidylethanolamine; PLT, platelet; S1P, Sphingosine-1-phosphate; S1PR1, S1P receptor 1; TG, triacylglycerols.

**Table 9:** Randomized clinical trials administering dietary interventions to improve ceramide concentrations in humans.

First Author	Origin	Design	Participants	Intervention(s)	Duration	Results
Airhart [179]	USA	DB, RCT	N = 16 patients with T2DM, an ejection fraction greater than 45% and no other systemic disease	(a) MCFA-rich diet containing 38% fat of the EI (b) LCFA-rich diet, containing 38% fat of the EI	14 days	The MCFA and not the LCFA diet reduced various plasma sphingolipids, ceramides and acylcarnitines implicated in diabetic cardiomyopathy. Changes in sphingolipids correlated with improved insulin.
Chen [180]	USA	RCT	N = 64 African-Americans with overweight/obesity	(a) 600 IU/day of vitamin D_3_ ONS (b) 2000 IU/day of vitamin D_3_ ONS (c) 4000 IU/day of vitamin D_3_ ONS (d) placebo ONS	16 weeks	Serum concentrations of *N*-stearoyl-sphingosine (d18:1/18:0) (C18Cer) and stearoyl sphingomyelin (d18:1/18:0) (C18SM) were significantly increased with vitamin D_3_ ONS, in a dose–response fashion. This was accompanied by correlations between serum 25(OH)D levels and these two metabolites.
Chiu [181]	USA	Cross-over RCT	N = 30 adolescent boys with overweight and obesity, all habitual consumers of sugar-sweetened beverages	(a) 24 oz/day of sugar-sweetened soda (*n* = 30) (b) an energy equivalent of reduced fat (2%) milk (*n* = 30)	3 weeks each arm, separated by a >2-week wash-out	Milk intake lowered plasma glucosyl Cer (d18:1/C16:0) and LactCer (d18:1/C16:0 and d18:1/C18:0). While no effects of replacing soda with milk on lipid and lipoprotein levels were observed in these normolipidemic weight-stable adolescent boys, decreases in SBP, UA and glycosphingolipids suggest an overall favorable effect on cardiometabolic risk can be achieved following a short-term dietary intervention.
Djekic [182]	Sweden	Cross-over RCT	N = 31 patients with CAD on standard medical therapy	(a) VD (*n* = 31) (b) isocaloric meat diet (*n* = 31)	4 weeks each arm separated by a 4-week wash-out	The VD intervention increased the levels of 11 TGs and lowered 21 glycerophospholipids, cholesteryl ester (18:0) and Cer (d18:1/16:0) compared with the meat diet. The VD also increased the circulating TGs with long-chain PUFA and lowered TGs with SFA, phosphatidylcholines and SMs.
Le Barz [183]	France	RCT	N = 58 postmenopausal women	(a) daily consumption of cream cheese with PL-enriched milk (3 g milk PL) (b) daily consumption of cream cheese with PL-enriched milk (5 g milk PL) (c) daily consumption of cream cheese without PL-enriched milk	4 weeks	Milk PL reduced serum atherogenic C24:1 Cer, C16:1 SM and C18:1 SM species. Changes in serum C16+18 SM species were positively correlated with the reduction in TC, LDL-C and ApoB. Milk PL decreased chylomicron content in total SM and C24:1 ceramides, parallel to a marked increase in total ceramides in feces.
Lindqvist [178]	Sweden	Cross-over RCT	N = 46 patients with RA	(a) MD (*n* = 46) (b) WD (*n* = 46)	10 weeks each, with a 4-month wash-out	No differences were noted in CERT2 after the MD compared with the WD, although several CERT2 components were improved.
Mathews [184]	USA	Parallel RCT	N = 36 young adults	(a) FRUVED (*n* = 12) (b) FRUVED + LRC (*n* = 12) (c) FRUVED + LF (*n* = 12)	8 weeks	The FRUVED intervention reduced circulating ceramides, including the C24:0 Cer. As inflammatory status improved with FRUVED, this was correlated with ceramide concentrations.
Rosqvist [185]	Sweden	DB, parallel RCT	N = 61 men and women with overweight or obesity	(a) muffins high in palm oil (SFA) added to the habitual diet (b) muffins high in sunflower oil (PUFA) added to the habitual diet	4 weeks of either arm, followed by 4 weeks of caloric restriction	SFA markedly increased liver fat and serum ceramide, whereas dietary PUFA prevented liver fat accumulation and reduced ceramides and hyperlipidemia in individuals with overweight.
Tuccinardi [186]	Italy	DB cross-over RCT	N = 20 healthy, normal-weight young subjects	(a) added chocolate spread [EI: 73% fat (EVOO), 20% CHO and 7% Pro], providing 570 kcal/d to an isocaloric diet (*n* = 20) (b) added chocolate spread [EI: 73% fat (palm oil), 20% CHO and 7% Pro], providing 570 kcal/d to an isocaloric diet (*n* = 20)	2 weeks	EVOO-enriched chocolate spread improved circulating sphingolipids and glucose profile, by reducing plasma cCer C16:0, Cer C16:0/Cer C22:0-Cer C24:0 ratio and SM C18:0.
Tuccinardi [187]	Italy	BD, cross-over RCT	N = 10 individuals with obesity	(a) a smoothie containing 48 g walnuts (*n* = 10) (b) a macronutrient-matched placebo smoothie without nuts (*n* = 10)	Twice each smoothie within 5 days, with a 1-month wash-out period between	The lipidomic analysis after the walnut smoothie showed a reduction in harmful ceramides, HexCers and SMs.
Wang [188]	Spain	RCT	N = 980 participants (*n* = 230 incident cases of CVD and *n* = 787 randomly selected participants with high CV risk, initially free from CVD diagnosis)	(a) MD supplemented with EVOO (*n* = 291) (b) MD supplemented with nuts (*n* = 262) (c) control diet (*n* = 234)	4.5 years	The Ceramide score was associated with a 2.18-fold greater risk of CVD. Participants with a higher ceramide score, assigned to an active intervention showed similar CVD risk to those with a lower score, whereas those with a higher ceramide score assigned to the control arm exhibited a greater CVD risk. Changes in ceramide concentrations were indifferent between MD and control arms during the first year.
Zhao [189]	China	RCT	N = 169 subjects with dyslipidemia	(a) placebo (*n* = 46) (b) 40 mg/day anthocyanins (*n* = 45) (c) 80 mg/day anthocyanins (*n* = 42) (d) 320 mg/day anthocyanins (*n* = 43)	12 weeks	Intake of dietary anthocyanins dose-dependently reduced plasma concentrations of all 6 ceramide species. Specifically, 320 mg/day of anthocyanins effectively lowered plasma *N*-palmitoyl sphingosine (Cer 16:0) and *N*-tetracosanoylsphingosine (Cer 24:0) compared with the placebo. The declines in plasma Cer 16:0 and Cer 24:0 were correlated with decreases in plasma non-HDL cholesterol.
Zhu [190]	USA	Cross-over RCT	N = 10 healthy individuals	(a) MD (*n* = 10) (b) fast-food diet (*n* = 10)	4 days each diet, with a 4-day wash-out between	The composition of PC, TG and CE were significantly altered to reflect the FA composition of the diet, whereas the composition of SM and ceramides were mainly unaffected.

25(OH)D, 25-hydroxyvitamin D_3_; ApoB, apolipoprotein B; Cer, ceramide; CAD, coronary artery disease; CE, cholesteryl ester; CHO, carbohydrate; CERT2, Cer- and phospholipid-based CVD risk score; CV, cardiovascular; CVD, cardiovascular disease; DB, double blind; EI, energy intake; EVOO, extra virgin olive oil; FA, fatty acid; FRUVED, high fruit and vegetables diet, equivalent to 2.5 or 3 cups of vegetables for women and men, respectively, and 2 cups of fruit daily for all individuals; HDL, high-density lipoprotein; HexCers, hexosylceramides; LactCers, lactosylceramides; LCFA, long-chain fatty acids; LDL-C, low density lipoprotein cholesterol; LF, low-fat; LRC, low refined carbohydrates; MCFA, medium-chain fatty acids; MD, Mediterranean diet; ONS, oral nutrient supplementation; PC, phosphatidylcholine; PL, polar lipids; Pro, protein; PUFA, poly-unsaturated fatty acyls; RA, rheumatoid arthritis; RCT, randomized controlled trial; SBP, systolic blood pressure; SFA, saturated fatty acyls; SM, sphingomyelin; T2DM, type 2 diabetes mellitus; TC, total cholesterol; TG, triacylglycerol; UA, uric acid; VD, lacto-ovo-vegetarian diet; WD, Western diet.

## Data Availability

Not applicable.
